# Different Methods of Dispersing Carbon Nanotubes in Epoxy Resin and Initial Evaluation of the Obtained Nanocomposite as a Matrix of Carbon Fiber Reinforced Laminate in Terms of Vibroacoustic Performance and Flammability

**DOI:** 10.3390/ma12182998

**Published:** 2019-09-16

**Authors:** Giuseppina Barra, Liberata Guadagno, Luigi Vertuccio, Bartolome Simonet, Bricio Santos, Mauro Zarrelli, Maurizio Arena, Massimo Viscardi

**Affiliations:** 1 Department of Industrial Engineering, University of Salerno, Via Giovanni Paolo II, 84084 Salerno, Italy; lguadagno@unisa.it (L.G.); lvertuccio@unisa.it (L.V.); 2 Nanotures, Jerez de la Frontera, 11400 Cadice, Spain; bartolome.simonet@nanotures.com (B.S.); bricio.santos@nanotures.com (B.S.); 3 Institute for Polymers, Composites and Biomaterials, National Research Council of Italy, P.le Fermi, 1, Portici, Naples 80055, Italy; mauro.zarrelli@cnr.it; 4 Department of Industrial Engineering, Aerospace Section, University of Naples “Federico II”, Via Claudio 21, 80125 Naples, Italy; maurizio.arena@unina.it (M.A.); massimo.viscardi@unina.it (M.V.)

**Keywords:** smart materials, carbon fiber reinforced polymers (CFRP), thermosetting resins, damping, sound transmission, heat release rate.

## Abstract

Different industrial mixing methods and some of their combinations ((1) ultrasound; (2) mechanical stirring; (3) by roller machine; (4) by gears machine; and (5) ultrasound radiation + high stirring) were investigated for incorporating multi-walled carbon nanotubes (MWCNT) into a resin based on an aeronautical epoxy precursor cured with diaminodiphenylsulfone (DDS). The effect of different parameters, ultrasound intensity, number of cycles, type of blade, and gear speed on the nanofiller dispersion were analyzed. The inclusion of the nanofiller in the resin causes a drastic increase in the viscosity, preventing the homogenization of the resin and a drastic increase in temperature in the zones closest to the ultrasound probe. To face these challenges, the application of high-speed agitation simultaneously with the application of ultrasonic radiation was applied. This allowed, on the one hand, a homogeneous dispersion, and on the other hand, an improvement of the dissipation of heat generated by ultrasonic radiation. The most efficient method was a combination of ultrasound radiation assisted by a high stirring method with the calendar, which was used for the preparation of a carbon fiber reinforced panel (CFRP). The manufactured panel was subjected to dynamic and vibroacoustic tests in order to characterize structural damping and sound transmission loss properties. Under both points of view, the new formulation demonstrated an improved efficiency with reference to a standard CFRP equivalent panel. In fact, for this panel, the estimated damping value was well above the average of the typical values representative of the carbon fiber laminates (generally less than 1%), and also a good vibroacoustic performance was detected as the nanotube based panel exhibited a higher sound transmission loss (STL) at low frequencies, in correspondence with the normal mode participation region. The manufactured panel was also characterized in terms of fire performance using a cone calorimeter and the results were compared to those obtained using a commercially available monocomponent RTM6 (Hexcel composites) epoxy aeronautic resin with the same process and the same fabric and lamination. Compared to the traditional RTM6 resin, the panel with the epoxy nanofilled resin exhibits a significant improvement in fire resistance properties both in terms of a delay in the ignition time and in terms of an increase in the thermal resistance of the material. Compared to the traditional panel, made in the same conditions as the RTM6 resin, the time of ignition of the nanotube-based panel increased by 31 seconds while for the same panel, the heat release rate at peak, the average heat release rate, and the total heat release decreased by 21.4%, 48.5%, and 15%, respectively. The improvement of the fire performance was attributed to the formation of a non-intumescent char due to the simultaneous presence of GPOSS and carbon nanotubes.

## 1. Introduction

In recent years, fiber reinforced polymer composites have grown exponentially in many industrial sectors such as automotive, aerospace, marine, and construction. There are several types of composites, and their quality depends on the structural and chemical properties of the raw materials used, as well as the process specifically employed for composite manufacturing [1,2,3,4,5,6,7,8,9,10,11]. Besides, the possibility to use carbon-based nanoparticles in thermoplastic or thermosetting matrices, combined with different processes, allows for achievement of enhanced mechanical, thermal, and electrical properties [6,12,13,14,15]. For instance, linear low-density polyethylene (LLDPE) nanocomposites with different percentages of multi-walled carbon nanotubes (MWCNTs) were prepared by microinjection molding; the resulting composites manifested improved strength and modulus together with enhanced toughness [14]. Among thermosetting matrices, the use of epoxy resins is increasingly growing. 

Carbon fiber reinforced composites (CFRCs), manufactured using epoxy matrices, have attracted considerable interest for the manufacturing of vehicle parts or load-bearing structures because of the mechanical resistance combined with the low weight, which in turn results in a strong reduction of fuel consumption and CO_2_ emissions. 

One of the strategies proposed in recent years to enhance the performance of CFRCs is based on the possibility to manufacture the CFRCs by impregnation of the carbon fabric with a resin containing incorporated nanostructured forms of carbon [5,6,8,12,16,17,18,19,20]. Nanofilled epoxy resins are unique with respect to other materials for their tailorability and the broad range of properties and related applications. They can be designed to have many distinct properties that may be exploited to develop the next generation of functional or self-responsive materials [21,22,23,24,25,26,27,28,29,30,31]. In particular, thermosetting resins filled with specific nanostructured particles can be manufactured to manifest enhanced mechanical, acoustic, electrical, and flame-resistance properties [25,32,33]. Furthermore, the incorporation of electrically conductive nanoparticles allows integration into the resin or carbon fiber reinforced panels (CFRPs) smart and self-protective functions, such as regenerative ability, self-sensing properties, anti/deicing, UV resistance, and possibly other functionalities that work in synergy to provide a new generation of structural–functional materials. 

The possibility to incorporate nanostructured forms of carbon, CNTs, nano-graphite, etc., in the resin can also help to improve the mechanical and adhesion performance of the resulting nanocomposites [34].

Among the nanostructured forms of carbon, unfunctionalized multi-wall carbon nanotubes (MWCNTs) are playing a very relevant role for their peculiar electrical properties. Epoxy resins filled with MWCNTs are able to reach the electrical percolation threshold (EPT) with very low percentages of nanoparticles. CFRPs manufactured using epoxy resins filled with MWCNTs exhibit high values of electrical conductivity. This peculiar property is strongly desired in aeronautics for lightning strike protection and electromagnetic characteristics. The possibility to disperse in the resin nanocages of GPOSS in combination with MWCNTs allows counterbalancing the increase in the viscosity of the resin, due to the presence of the nanotubes, simultaneously conferring to the nanofilled resin flame resistance property and self-healing ability [29].

Furthermore, CFRPs manufactured using this combination of additives, CNTs, and GPOSS highlighted a significant decrease in the fatigue crack growth rate of about 80% [35]. 

This result can help to design composite materials able to fulfill some of the strongly desired requirements for their application in aeronautics, which is a very complex study. In fact, the development of a carbon fiber reinforced composite (CFRC) and the preservation of its structural integrity requires the investigation and control of different interacting factors: critical aspects concerning the application, accessibility, and ability for the inspection of vital parts and components; studies on the consequences of impact, fatigue, temperature, and hostile environment; nature of inherent flaws; etc. [36]. Capezzuto et al. proposed an interesting strategy for the detection of low-velocity impact damage on composite structures [37]. The study of this aspect is of relevant interest because damages due to low-velocity impact events not only weaken the structure undergoing a continuous service load but also may generate different types of flaws before full perforation, i.e., sub-surface delamination, matrix cracks, fiber debonding or fracture, indentation, and barely visible impact damage (BVID) [37]. The possibility to confer the ability to decrease fatigue crack growth rates in CFRCs is a current challenge that may be solved using a combination of CNTs and GPOSS [35].

However, one of the most difficult challenges to face when CNTs are incorporated in epoxy resins is related to the difficulty of obtaining acceptable levels of dispersion. In fact, carbon nanotubes tend to form bundles because of intense intertubular van der Waals attractive forces, which can prevent obtainment of materials with high reproducibility in all the microzones of the bulk material. Hence, a dispersion assessment is of primary importance for the manufacturing process of composites [8,9,10]. The work described in this paper regards the manufacturing processing of coupons of epoxy multifunctional composites based on carbon fiber reinforced epoxy resin nanofilled with CNTs. More specifically a study of the optimization of the dispersion methods was performed.

The crucial stage of nanoparticle dispersion was studied through a series of experiments. In particular, several mixing methods and some of their combinations, namely (1) ultrasound; (2) stirring; (3) by roller machine; (4) by gears machine; and (5) ultrasound radiation + high stirring were investigated to disperse MWCNT in an epoxy formulation containing solubilized GPOSS nanocages. The chemical composition of the epoxy matrix was chosen to obtain high mechanical performance suitable for manufacturing load-bearing structures. 

It is well known that the combination of epoxy resin and hardener defines the matrix material properties and the various possible combinations allow one to tailor material properties according to the desired requirements [1]. In this work, the tetrafunctional epoxy precursor TGMDA was used in combination with a reactive diluent to decrease the viscosity of the resin and facilitate the step of nanofiller dispersion in the matrix; 4,4 diamminodioheynil sulfone (DDS) was used as a hardener agent due to the high mechanical performance of the resin solidified with this class of curing agent. 

## 2. Materials and Methods 

### 2.1. Materials

Epoxy resin. The epoxy matrix was prepared by mixing the epoxy precursor TGMDA (epoxy equivalent weight 117–133 g/eq) with the epoxy reactive monomer 1,4-butanedioldiglycidylether (BDE) that acted as a reactive diluent. These resins, both containing epoxy functionality, were obtained from Sigma-Aldrich. The epoxy mixture was made by mixing TGMDA with BDE monomer at a concentration ratio of 75:25 wt% epoxide to flexibilizer. In particular, the use of a percentage of 25% of reactive diluent was chosen to reduce the viscosity of the epoxy resin and hence to improve the nanofiller dispersion. 

Carbon nanotubes. The MWCNTs (3100 grade) were obtained from Nanocyl S.A (Sambreville, Belgium). The average diameter and the average length, evaluated by high-resolution transmission electron microscopy (HRTEM), were 9.5 × 10^−9^ m and 1.5 μm, respectively. In particular, an outer diameter ranging from the minimum of 10 nm to the maximum of 30 nm was measured, whereas the length of MWCNTs was from hundreds of nm to a few micrometers. The number of walls varied from 4 to 20 in most nanotubes. The specific surface area of MWCNTs estimated by the Brunauer Emmett Teller (BET) method was around 250–300 m^2^/g; The carbon purity and the metal oxide percentage, calculated by thermogravimetric analysis (TGA) were >95.0 and <5.0, respectively. An amount of 0.5 wt% of MWCNT was used for blend preparation.

POSS molecules. Glycidyl oligomeric silsesquioxanes (GPOSS), functionalized with eight oxirane groups for each molecule, were dispersed in the epoxy matrix. The POSS/epoxy composites were prepared with 5 wt% of POSS. GPOSS was purchased from Hybrid Plastic (USA).

Curing agent. DDS (4,4′ diaminodiphenyl sulfone), purchased from Sigma-Aldrich (Milan, Italy) was used as a hardener agent and added at a stoichiometric concentration with respect to all the epoxy rings arising from TGMDA, BDE, and POSS.

Carbon fibers. Thermofixed unidirectional carbon fabric was used for the preparation of the composites. In particular, thermofixed UD carbon–GV 501 U TFX (G. Angeloni s.r.l., Quarto d’Altino (VE), Italy), with an areal density of 0.516 kg/m^2^ and fibers composed of 24,000 individual carbon filaments, was used. The thickness was 0.5 mm.

### 2.2. Methods

#### 2.2.1. Study of the Dispersion of Carbon Nanotubes in the Epoxy Matrix and Panel Preparation

For the study of the dispersion of carbon nanotubes, different techniques (ultrasonication, mechanical stirring, gearbox milling, and calendaring (three-roll mill) were considered. The use of the combination of the different techniques, such as ultrasound-assisted with high stirring, or combining, for instance, the ultrasound-assisted with a high stirring method with the calendaring, were also taken into account. 

Ultrasonication was performed using an ultrasonic device, Hielscher model UP200S (200 W, 24 kHz) (Hielscher Ultrasonics, Teltow, Germany). Two different sonotrodes were used according to the volume of the dispersion: a 3 mm tip sonotrode for volumes from 5 mL up to 200 mL and a 22 mm tip sonotrode for volumes from 100 mL up to 1000 mL.

**Figure 1:**
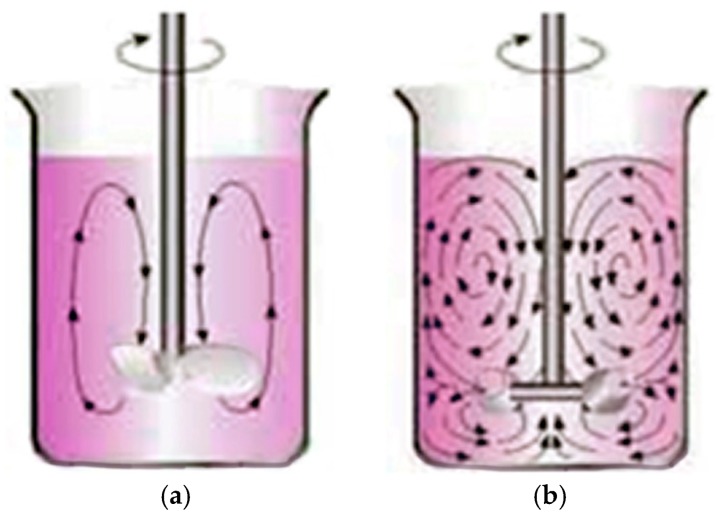
Flows generated by using a helix blade stir bar (**a**) and viscojet stir bar (**b**).

A Heidolph RZR-2102 stirrer (Schwabach, Germany) was used for the mechanical stirring. Three different mixing elements were used in order to evaluate the effect of the flow generated by the impeller on the particle dispersion: a helix blade, a viscojet with two cones, and a viscojet with three cones. The main difference between a helix blade stir bar and a viscojet is the flow generated into the matrix, and therefore the efficiency in the dissipation of heat (Figure 1).

**Figure 2:**
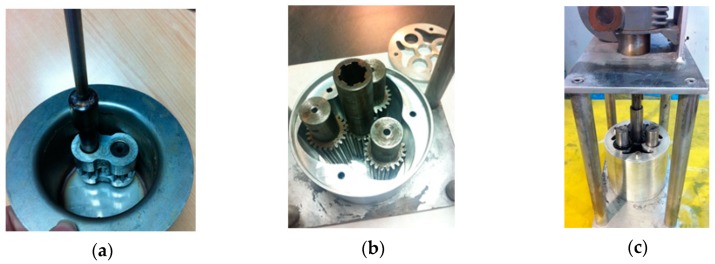
In house assembled gearbox miller; the two gear head is observable in (**a**) and three gear head from two different perspectives are observable in (**b**) and (**c**).

The gearbox miller was made and assembled in house as shown in Figure 2. 

The roller machine (SDW800) was purchased from Bühler S.p.A Segrate (Milan) Italy.

The dispersion degree of the MWCNT in the epoxy resin on the microscale was investigated with an optical microscope (Olympus BX51, Tokyo, Japan). Three images were obtained for each material type, and these were used for the analysis. Although this method is a low-accuracy method and rudimentary, the reliability and measurement accuracy of aggregate size is sufficient to decide which method had the best dispersion of nanofillers and to measure the average size of the agglomerates of particles in the system in question.

Standard conditions set up in all dispersion methods were used for the dispersion of the filler. Many values of the setting parameters were analyzed in order to obtain a wide range of data, allowing a representative comparison between all methods and the optimization of the process. 

#### 2.2.2. Preparation of the Epoxy Resin

A master batch (1 kg) of epoxy nanofilled resin was prepared in order to manufacture a laminate. DDS in a stoichiometric amount with respect to the total oxirane rings was added to the epoxy precursor blend constituted by TGMDA 75 wt% and BDE 25 wt% at 120 °C until complete hardener solubilization. Then the mixture was cooled to 90 °C. At this temperature, an amount of carbon nanotubes and GPOSS compounds, equal to 0.5% and 5% in weight of the amount of the epoxy precursor blend, respectively, were added using the dispersion method providing the best dispersion results (see Section 3.1. Efficiency of dispersion methods).

#### 2.2.3. Laminate Manufacturing 

Flat panels were prepared using the epoxy matrix containing carbon nanotubes. A proper amount of resin was used in order to obtain a panel with a 50:50 fiber to matrix ratio. 

The manufacturing process consisted of three main steps:

- Pre-impregnation
- Hand lay-up of the prepreg and preparation of the bag vacuum molding
- Curing in autoclave

A prepreg laminate was firstly prepared to ensure even resin distribution, avoiding the dry spots and resin-rich pockets common with infusion processes. The feeding of materials in the equipment used for the pre-impregnation process was carefully done so that the fabric was perfectly aligned, and the resin was homogeneously distributed.

At this stage, the alignment of the pressure rollers, designed to evenly distribute the resin by the pressure exerted on the fabric, is a crucial aspect. After feeding the material for prepreg manufacturing, the process continues automatically through a series of several rolls to give the prepreg previously studied. 

For the hand lay-up of the prepreg, the release agent, Marbocote (UK), was applied to the mold. A laminate 0/90/0/90/0/90 was prepared with a tolerance of ±5° on the orientation. During the placement of fabrics, air entrapment and wrinkling were avoided by applying pressure to the fibers in the warp direction. Then, the vacuum bag was prepared according to the following scheme:

The bag was kept under vacuum until the internal pressure was between 0.1 and 0.8 bar (76–610 mm Hg) before its introduction into the autoclave for the curing cycle.

**Figure 3:**
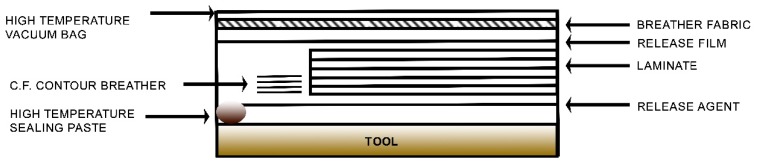
Scheme of the prepared vacuum bag.

The curing conditions used an initial step at moderate temperature (125 °C for 1 h) followed by a second one at higher temperature (180 °C for 3 h). Figure 3 shows a graphical registered autoclave cycle for a manufacturing demonstrator.

**Figure 4:**
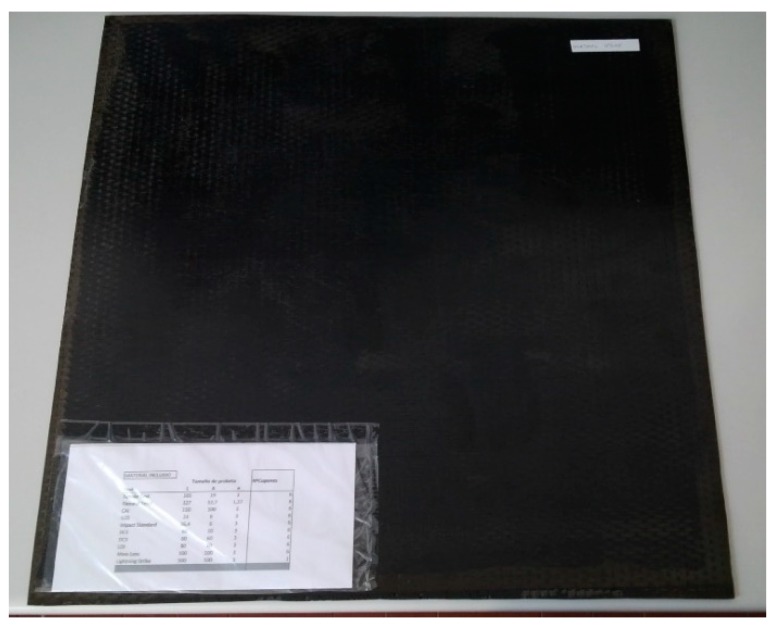
Picture of the manufactured panel.

A picture of one of the prepared panels is shown in Figure 4.

### 2.3. Characterization Methods

#### 2.3.1. Laser Scanning Vibrometry Test

**Figure 5:**
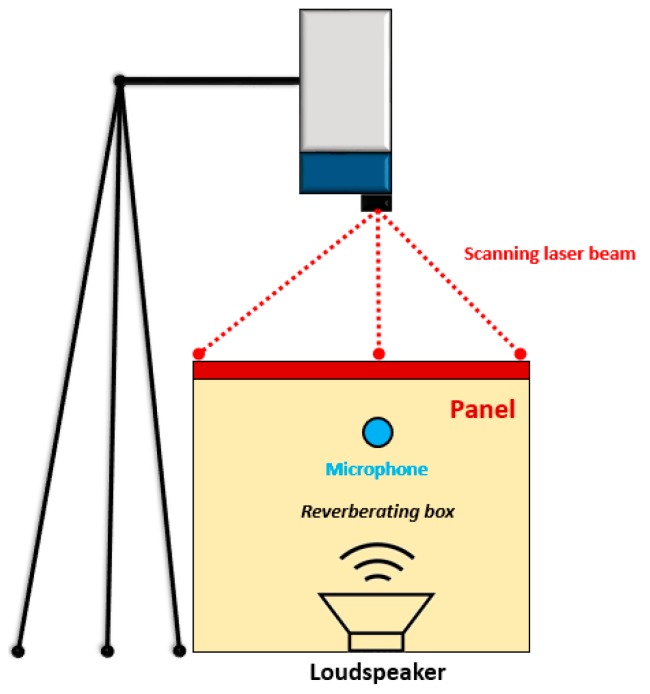
Laser vibrometry test set-up.

The main purpose of the vibrometry test was to estimate the operative deflection shapes (ODS) that under the white noise excitation condition traduced into the determination of modal frequencies and relative damping coefficients. The test facility consisted of a reverberation box in which a speaker served as an acoustic loading element of the panel, which was simply-supported on the four edges of the box (Figure 5). These edges were bonded on soft material sheets (i.e., polystyrene) in order to avoid any coupling mechanism among the plunge rigid motion and the interested elastic mode shapes, and at once avoiding acoustic energy losses. The presence of a microphone positioned in proximity to the sample served to measure the sound pressure level (SPL) of the incident sound waves. Next, to the outlet surface, a scanning laser head (Polytec PSV 400) was positioned to measure the vibration velocity of the test article. The whole measurement chain was characterized by a class 1 level.

At the base of this acquisition, there is a dense theoretical background that permits simulating the vibrating behavior of the samples, particularly its own natural frequencies. Specifically, under well-defined constrained conditions, i.e., a simply-supported sample, the empirical formula for calculating these frequencies is as follows:

(1) $$\omega r=\sqrt{\frac{D}{m}}\left[{\left(\frac{r_{1}\pi }{a}\right)}^{2}+{\left(\frac{r_{2}\pi }{b}\right)}^{2}\right],$$

where *r_1_* and *r_2_* are the modal indices of its modes *r^th^*, *m* is the mass per unit of area, *D* the bending stiffness, and *a* and *b* are the dimensional quantities of the sample (length and thickness, respectively). Furthermore, it is also possible to calculate the critical or resonance frequency of the single sample by means of a second relationship, a function of the speed of sound *c* (340 m/s) and of the previous parameters:

(2) $$\omega_{c}=c^{2}\sqrt{\frac{m}{D}}$$

Furthermore, associated with these formulas, the law for its associated modes calculation is defined as:

(3) $$\varphi_{r}(x,y)=2\sin\left(\frac{r_{1}\pi x}{a}\right)\sin\left(\frac{r_{1}\pi y}{b}\right)$$

#### 2.3.2. Sound Transmission Loss Test

**Figure 6:**
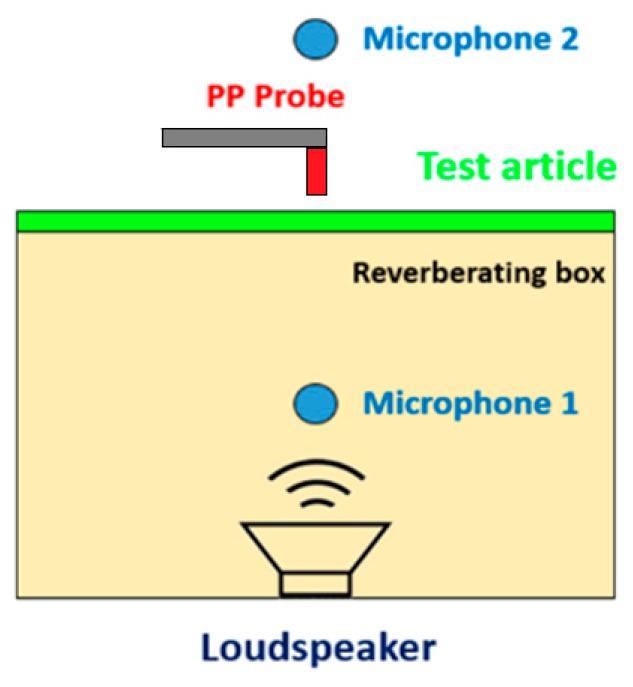
Experimental layout for STL measurement of engine cover based on sound intensity method.

Sound reduction index (SRI) or sound transmission loss (STL) is the most usual product-related acoustical quantity determined in laboratory or field conditions. The sound insulation performance in terms of sound transmission loss (STL) was assessed according to the ISO 15186 standard, because of the dimension of the test article. Figure 6 shows a schematization of the experimental layout. In this case, a broadband sound source was always placed in a reverberating box and the panel positioned as in the laser scanning test. Taking into account the general expression for STL (Equation (4)) and according to the reference standard, the STL was measured as a difference of the incident sound field (because incident SPL in a reverberating environment is directly related to the incident sound power) and the transmitted sound intensity (because also the spatial integration of sound intensity is directly correlated to the transmitted sound power).

(4) $$STL=10\log_{10}\left(\frac{1}{\tau }\right),\;\mathrm{where}\;\tau =\frac{W_{t}}{W_{i}}$$

Hence, the general equation for STL evaluation under the ISO 15186 hypothesis can be written as:

*STL* = *L_p_* − *L_i_* − 6 (5)

where *L_p_* is the average sound pressure level inside the box and *L_i_* is the sound intensity level over the measurement surface.

This standard is, in fact, very useful when avoiding the use of the double chamber method is necessary. 

#### 2.3.3. Cone Calorimeter Test

Combustion studies were performed by using an oxygen consumption calorimeter (Fire Testing Technology, FFT dual cone calorimeter model). The rectangular samples 10 × 10 × 0.5 cm^3^ were irradiated at a heat flux of 35 and 50 kW/m^2^. Three replications were performed at each irradiance and the results are shown as the average of the curves. Data obtained on the panels manufactured with the nanofilled resin were compared to those obtained using a commercially available monocomponent RTM6 (Hexcel composites) epoxy aeronautic resin with the same process and the same fabric and lamination.

## 3. Results and Discussion

### 3.1. Efficiency of Dispersion Methods

The influence of the different techniques at different conditions on the size of the aggregate was analyzed:

#### 3.1.1. Effect of Ultrasound Intensity at a Pulse Cycle of 75% and Ultrasound-Assisted with High-Speed Mechanical Agitation

**Figure 7:**
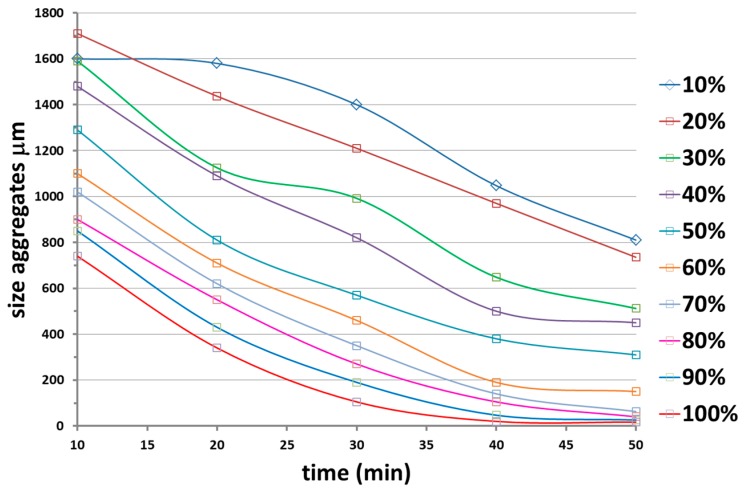
Effect of the ultrasound intensity at constant pulse of 75% on the size aggregation: evolution with the time of the size aggregates when ultrasound is applied at intensities ranging from 10 to 100%.

The effect of the ultrasound intensity at the constant pulse of 75% on the size aggregation is shown in Figure 7. The size of the aggregates was monitored in the time when ultrasounds were applied at different intensities ranging from 10% to 100%. It is evident that the minimum particle size was obtained using the maximum intensity of 100%.

**Figure 8:**
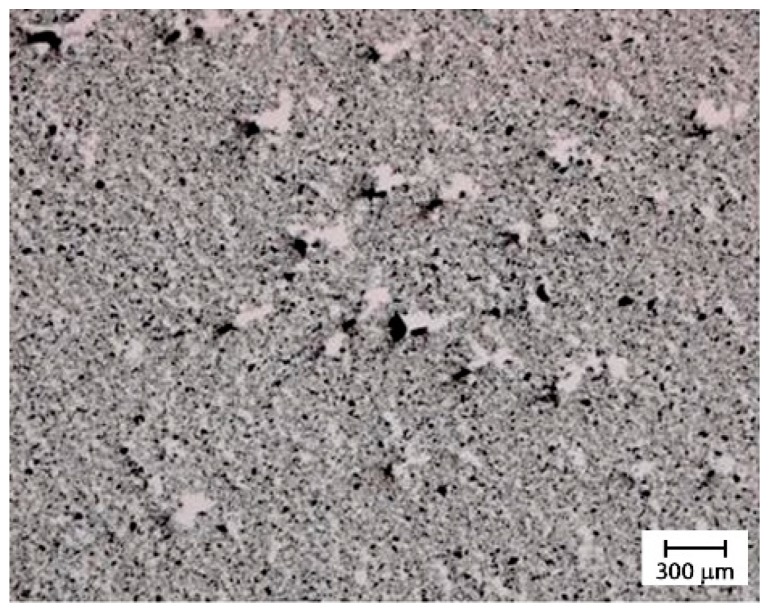
Optical micrograph of the dispersion obtained applying ultrasounds at 100% intensity for 30 min.

Figure 8 shows as example an optical microscopy picture made for the determination of the size aggregates.

The main advantages of this method are the simplicity, the feasibility, and the inexpensiveness with respect to other methods, although it has some drawbacks related to the excessive heating experienced by the matrix when applying the ultrasonic radiation, particularly in the zones closest to the ultrasound probe. 

This heating produces two competitive effects on the dispersion:

- decreases in the viscosity of the resin matrix; this effect facilitates the nanofiller dispersion within the matrix and allows a better homogeneity of the nanofiller dispersion.
- re-agglomeration of the dispersed nanoparticles caused by the excessive increase in temperature of the matrix for extended periods of time.

Therefore, the choice of the conditions is very important to obtain viscosity low enough to facilitate the dispersion and the homogenization of the fillers without any re-agglomeration effect.

One of the possibilities of overcoming the heating issues is the simultaneous application of high-speed agitation with the ultrasound. 

This allows one to obtain a more homogeneous dispersion as an effect of the additional agitation together with an improvement of the dissipation of heat generated by the ultrasonic radiation.

**Figure 9:**
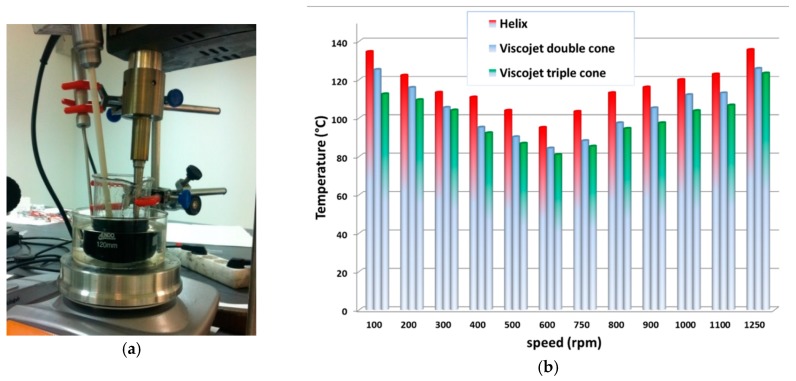
(**a**) Ultrasound-assisted with high-speed mechanical agitation set up; (**b**) effect of the applied agitation speed of three different probes on the temperature: (1) helix, (2) viscoject double cone, (3) viscoject triple cone.

Figure 9a shows a picture of the experimental setup. It is possible to see the ultrasound probe, adapted to the volume and the intensity of the applied radiation, the stripping shovel, whose speed depends on the viscosity of the matrix, and a thermostatic bath to maintain temperature equilibrium. The ultrasound was set at cycle pulse = 75% and intensity = 100%, and the type of probe and the high stirring speed was varied. Figure 9b shows the effect on the temperature of the applied speed for any of the analyzed probes. It is evident that the higher heat dissipation was obtained using the viscojet triple cone at 600 rpm. 

The best conditions for the ultrasound-assisted with high-speed mechanical agitation were obtained when ultrasound with pulse = 75% and intensity = 100% was applied together with the viscojet triple cone at 600 rpm.

#### 3.1.2. Effect of the Gear Speed

**Figure 10:**
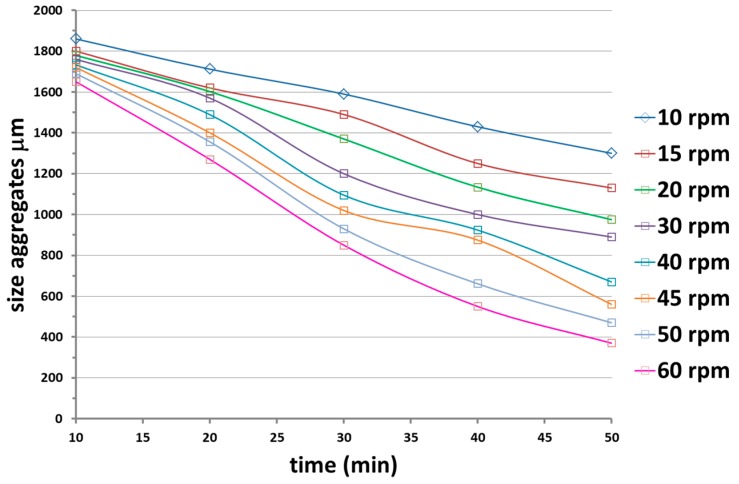
Evolution of the size aggregates with the time of application at different gears speed for a two gear head gearbox miller.

**Figure 11:**
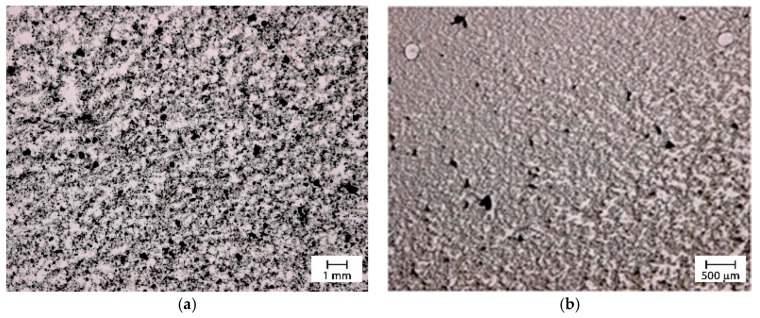
Optical micrographs of the dispersion obtained applying gear speed at 60 rpm for 60 min, (**a**) two gear head (**b**) three gear head.

The effect of the gear at speeds ranging between 10 and 60 rpm was analyzed. Figure 10 shows the evolution with the time of the size aggregates at the different gear speeds for a two gear head gearbox miller. Unfortunately, with respect to the previous methods, no improvement on the quality of the dispersion was observed. The optical microscopy images in Figure 11 are related to the dispersion obtained using the two gear head (a) and the three gear head (b) at 60 rpm. Although the 3 gear head gearbox milling provided the best results this was not good enough to obtain an optimal dispersion.

#### 3.1.3. Calendar: Effect of the Roller Distance

**Figure 12:**
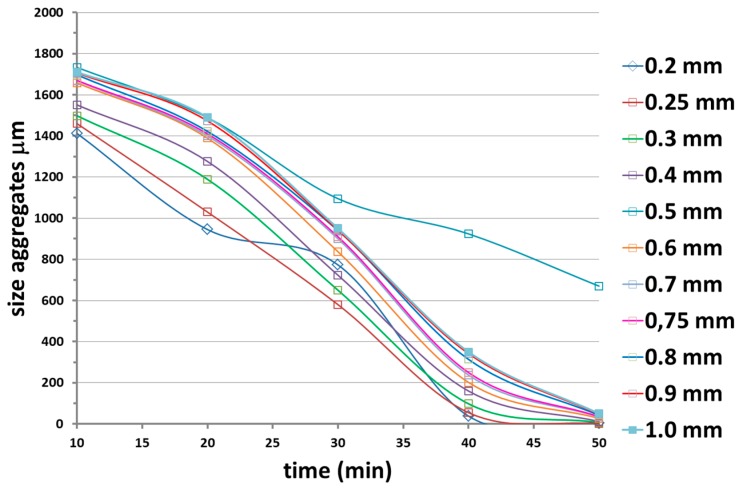
Evolution of the size aggregates with the time of application at different distances between the rollers.

This mechanical dispersion method used a pressure roller machine. The distance between the rollers, which was of the order of microns, was varied in order to optimize the process of nanofiller dispersion.This method, offering the advantage of constantly driving the nanofilled matrix through the rollers, was highly efficient. The temperature of the rollers was set at 90 °C. Figure 12 shows the evolution with the time of the size aggregates at different distances between the rollers

**Figure 13:**
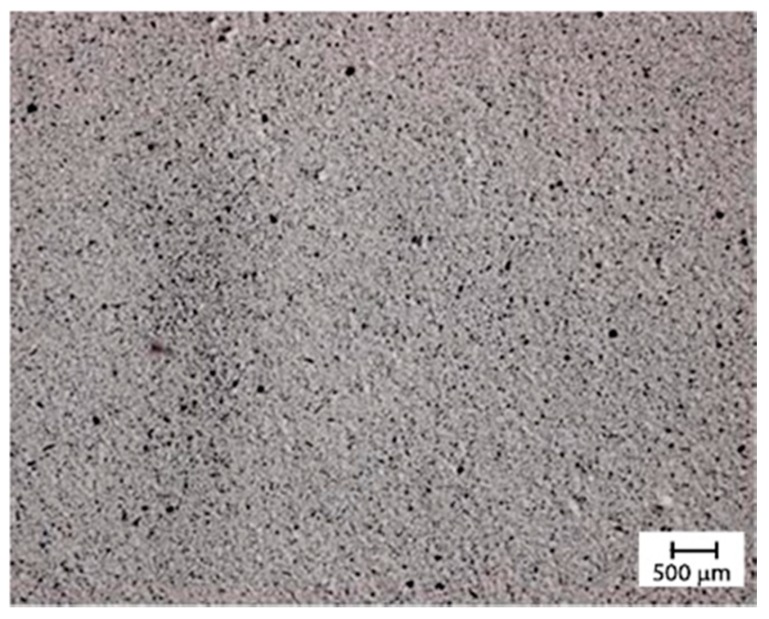
Optical microscopy of the dispersion obtained with the calendar with a distance between the rollers of 0.25 mm.

Figure 13 shows the optical microscopy of the dispersion obtained with the calendar set with a distance between the rollers of 0.25 mm. The predominant grey color with few very little white and black spots indicates the good quality of this dispersion method.

The parameter to optimize was the distance between rollers, which was studied in a larger range of values. This study highlighted that the optimal distance between the rollers was 0.25 mm. 

#### 3.1.4. Effect of the Mixed Method (2 STEPS)

The mixed-method was a combination of two of the previous methods. Concretely, it consisted of a first step “Ultrasound radiation assisted by high stirring method” at cyclic pulse of 75%, the intensity of 100% for the ultrasound, and of the use of the viscojet triple cone at 600 rpm for the mechanical high speed stirring, followed by a second step with the calendar for which the distance between rollers was set at 2.5 mm.

**Figure 14:**
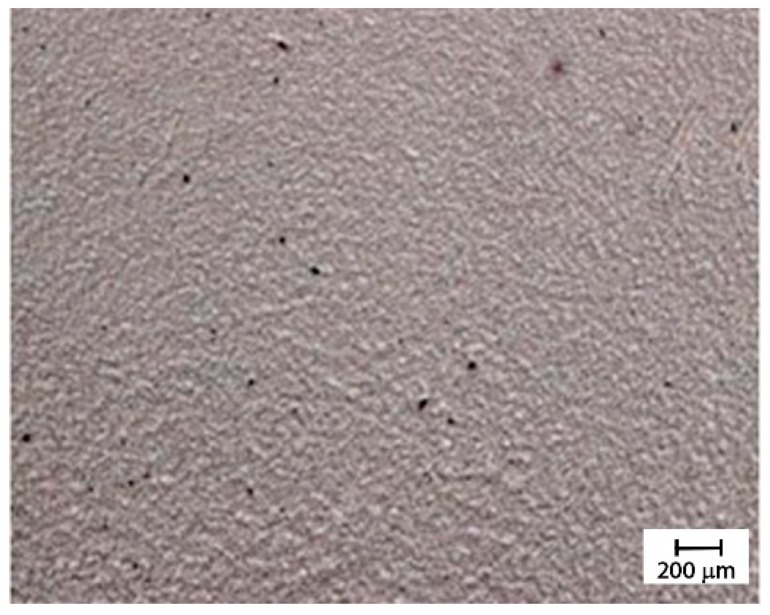
Optical micrograph of the dispersion obtained with the mixed method.

With respect to all the previous methods, this combined method resulted in a nanofilled material with a better dispersion and homogeneity. This was confirmed by the optical micrograph shown in Figure 14, which referred to a dispersion obtained in such a condition in which the sample was almost entirely grey. No white spots were evident and only a negligible amount of black spots indicating the presence of nanofillers agglomerate was observable.

### 3.2. Structural Damping and Acoustic Performance Assessment 

**Figure 15:**
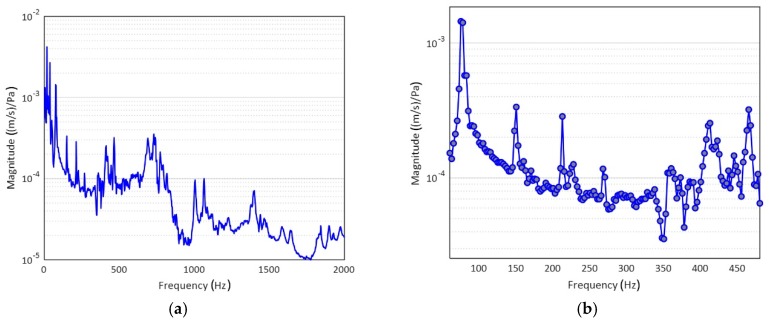
Laser vibrometry results, Frequency Response Functions (FRF) between surface vibration and acoustic pressure: (**a**) broad-spectrum analysis; (**b**) spectral windowing of modal region.

**Figure 16:**
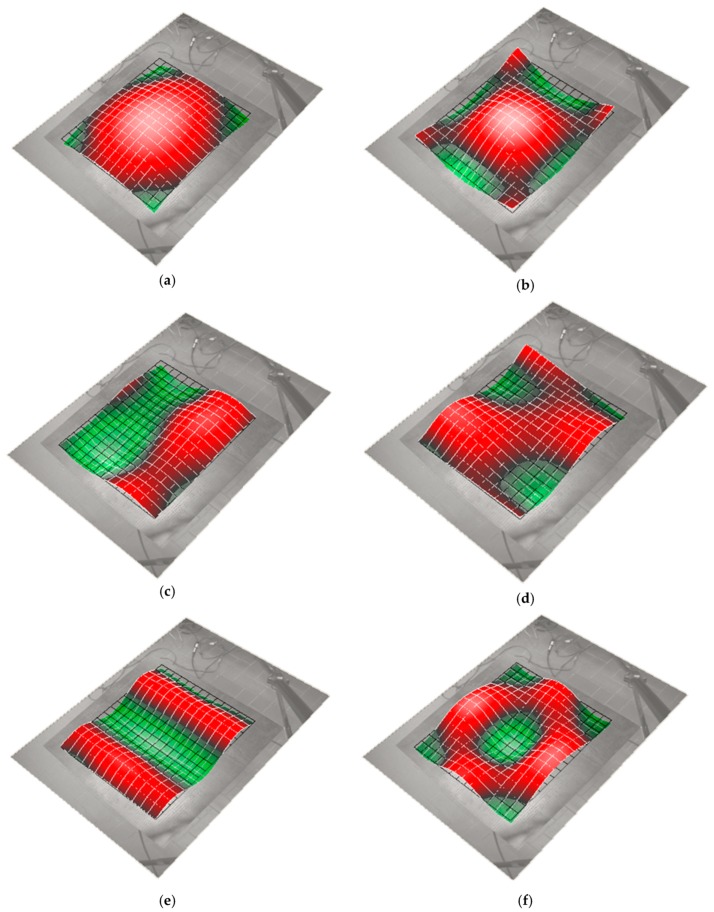
Operating deflection shapes: (**a**) tympanum mode, f = 80 Hz; (**b**) first symmetric bending mode, f = 150 Hz; (**c**) first anti-symmetric bending mode, f = 213 Hz; (**d**) second symmetric bending mode, f = 228 Hz; (**e**) third symmetric bending mode, f = 410 Hz; (**f**) fourth symmetric bending mode, f = 465 Hz.

The laser tests carried out on the examined sample brought about a series of important results regarding the modal-vibrational behavior, with respect to acoustic excitation in the frequency range between 0 and 2000 Hz. These results were then implemented and post-processed in a Matlab^®^ environment: the frequency response (vibration velocity/input pressure level), shown in Figure 15, presented a series of peaks, which represented a measure of the modal vibrations. Specifically, the peaks could be associated with defined resonance frequencies, typical of the panel modes, and depending on these peaks, it was possible to extract the specimen’s damping coefficient for that specific mode. In such a way, it was possible to quantify an important acoustic property of the analyzed material: the ability to limit its deformations under the action of a relevant pressure load. Excluding the initial zone with the maximum peaks, several resonances were present in the spectral range between 80 and 480 Hz (Figure 15b). The presence of such maximums allowed us to observe how the most intense modes were recorded for relatively low frequencies, compared to the much higher range of pure acoustic application. Each natural frequency corresponded to a proper vibrating mode shape: in Figure 16, six different elastic modes well captured in the interval 80–480 Hz are represented.

Relying upon the transfer function represented above, the damping coefficient for the examined modes was estimated. Naturally, the damping evaluation was based on a very precise spectral method, albeit rather elementary, which is the half-power method: it consists of taking the "peaks" of interest into consideration, thus corresponding with their modes; the peak’s frequency value is considered below, which is nothing other than the proper frequency of the mode (φ_0_), and two higher and lower frequency values, respectively, that function as reference values for an average estimate of the damping (φ_2_ and φ_1_), as described in the formula:

(6) $$\zeta =\frac{\varphi_{2}-\varphi_{1}}{2\varphi_{0}}.$$

The reference values can easily be estimated by interpolating the panel’s frequency response functions (FRF)-average curve in the peak’s frequency interval with a value line ordinated by the peak related by the root of two:

(7) $$A_{rif}=\frac{A_{max}}{\sqrt{2}}.$$

**Figure 17:**
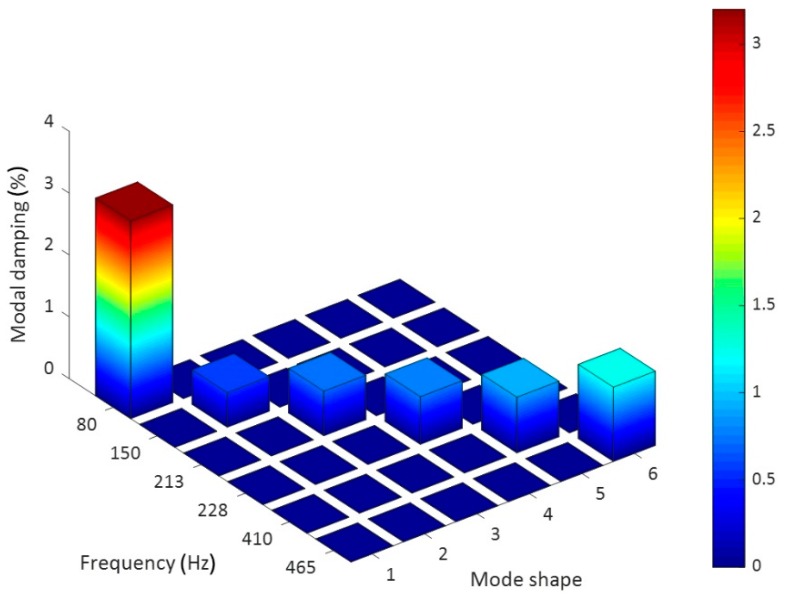
Modal structural damping for the six elastic mode shapes.

The results achieved through the previous method were tabulated and diagrammed to make comprehension better (Figure 17).

The dynamic analysis in the spectral domain highlighted a high dissipating property of the analyzed material mostly in the low-frequency region. The estimated damping value was well above the average of the typical values representative of the carbon fiber laminates (generally less than 1%). The result is encouraging because the highest damping coefficient was found in the correspondence of the "drum" mode (0,1) where the maximum modal participation of the structure was obtained. Moreover, it also represents a very relevant mode from the vibro–acoustic standpoint, as it is characteristic of an emissive membrane mode. Such a (0,1) planar mode is excited by impacts at any location on the membrane. When vibrating in this mode, the membrane behavior is much like a monopole source, which radiates sound very effectively. Since it radiates sound so well when vibrating in this manner, the membrane quickly transfers its vibrational energy into radiated sound energy and the vibration dies away. 

In addition to the numerous known applications of CNTs in composite materials, in order to improve their mechanical properties, in recent decades research tackled the analysis of the acoustic properties of these carbon structures to exploit their application in many industrial fields, in particular automotive and avionics, with the aim of amplifying the insulating characteristics of various components of aircraft and vehicles. In view of this function, the study also focused on the assessment of these insulating capacities.

**Figure 18:**
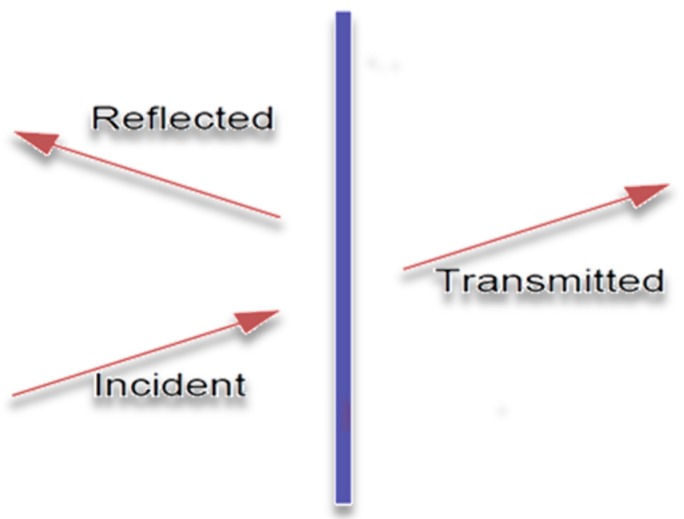
Sound power transmission diagram.

In fact, a material, if stimulated by an acoustic load or by a simple sound wave with an assigned acoustic power will tend to pass only part of it, according to its chemical structure and its nature: specifically, the initial power ωi will be divided into three aliquots (Figure 18).

(8) $$\omega_{i}=\omega_{a}+\omega_{r}+\omega_{t},$$

where the contributions are, respectively: the absorbed power, the reflected power, and the passing power (even if we often tend to sum the powers absorbed and reflected in a single contribution); the relationship can easily be redefined as adimensional:

(9) $$1=\alpha +r+\tau \;:\;\{\begin{array}{c} \alpha =\frac{\omega_{a}}{\omega_{i}}\\ r=\frac{\omega_{r}}{\omega_{i}}\\ \tau =\frac{\omega_{t}}{\omega_{i}} \end{array},$$

**Figure 19:**
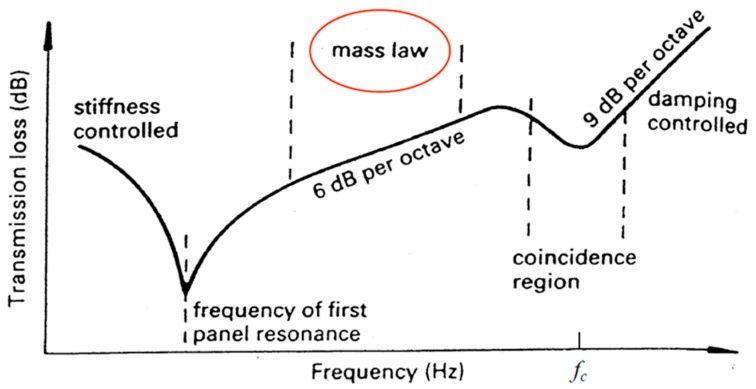
Classic transmission loss (TL) scheme according to frequency.

In the present analysis, it was interesting to understand how the CNTs panel could reflect and absorb the acoustic power of the sound source, measuring the transmission loss as previously already introduced. A curve that illustrates the variation of the TL as a function of the frequency associated with the acoustic source was graphically obtained, as in the example in Figure 19.

The graph model, above illustrated in Figure 19, is a general scheme of the transmission loss (TL) of material and presents the classical decreasing–increasing trend for low-frequency range up to the “linear” section regulated by the Law of the Mass. This law is essential to analytically define the TL respecting known parameters:

(10) $$TL=20\mathrm{Log}\left(\frac{\omega \mu }{2\rho_{0}c_{0}}\right).$$

The term in the denominator is the specific impedance characteristic, while the contributions to the numerator depend on the nature of the sample being analyzed, i.e., a panel hit by plane waves.

**Figure 20:**
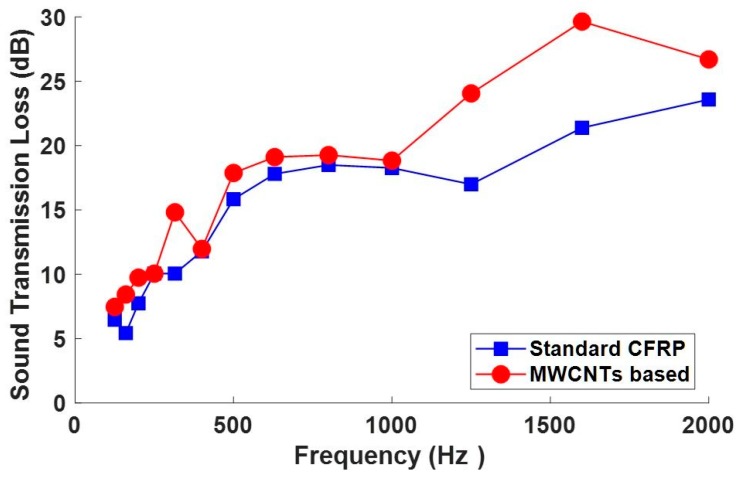
Transmission Loss. CFRP is carbon fiber reinforced panel.

The measured sound transmission loss of the manufactured panel and the STL of a standard CFRP panel are reported in Figure 20. 

The sound insulation performance comparison was performed with respect to other literature data on similar panel configurations, due to the absence of a reference standard formulation. Koval [38], performed deep studies in 1980 on the transmission of airborne noise into an aircraft cabin: results demonstrated that the noise attenuation provided by a composite shell was not so advantageous as an aluminum wall, mainly due to the increased acoustic radiation efficiency of CFRP. The graph above (Figure 20) shows then a good performance of the nanotube based panel exhibiting already a higher STL at low frequencies, in correspondence with the normal mode participation region. Such behavior can be explained by the modal damping peculiarities of the panel as previously measured. It can be, in fact, assumed that the resonant path for STL is attributed to the coupling of acoustic waves to free bending waves in the panel. It usually dominates the overall response around and above the panel coincidence frequency, where the acoustic wavelength is about the same as the structural wavelength, making the panel radiation more effective. In this frequency range, it is the damping loss factor that primarily controls panel vibration response and consequently the sound transmitted through the panel, so the higher the loss factor, the higher the STL [39,40]. Moreover, an interesting value of the STL increasing up to 30 dB is then measured in the medium frequencies range, next to 1500 Hz.

This result seems to be of general validity. In fact, the obtained results confirm what has already been published on specimens obtained with the same formulations but using ultrasound as a dispersion method and a modified liquid resin infusion technology for the manufacturing of the panels [26]. 

In particular, the authors compared the results obtained for little coupons of CFRP impregnated with filled and unfilled epoxy resin. The obtained results indicated that reinforcing a sample with carbon structured nanofillers positively affect the properties of the sample since they, de facto, contribute to improving the global structural performance and to simultaneously positively enhancing the modal damping determined both in the time and in the spectral domains.

In general, damping in composites is caused by the viscoelastic behavior of the polymer matrix [41]. Although the load is transferred mainly by fibers, the matrix takes part in its transmission by means of tangential stresses [42,43,44]. In [26] the authors also showed dynamic mechanical characterization (DMA) of an unreinforced cured coupon made with the same formulation used in this paper and compared the results with those obtained with the same unfilled formulation taken as reference. While in the range of temperature between 180 and 300 °C, only one peak centered at 260 °C was observable in the tan δ spectrum of the unfilled formulation, the tan δ spectrum of the formulation containing both GPOSS and CNTs in the same range of temperature was characterized by the presence of two distinct peaks: the first centered at 210 °C and the second centered at 260 °C. Other published works [27,29] demonstrated that in these systems the presence of MWCNT was responsible for a greater mobility of chains belonging to domains finely interpenetrated in the matrix where reversible hydrogen bonds are determined by interaction between epoxy resin and nanocages of POSS compounds. In particular, the two peaks were attributed to the presence of two phases with different cross-linking density and hence with different chain mobility. It was evident that the nanofiller was exerting a strong influence on the structure of the matrix. The different cross-linking density was ascribed to an interruption of the cross-linking reactions on the part of CNTs during the curing cycle and related to the decrease of curing degree. The result was the formation of a fraction of the resin with a lower Tg characterized by greater mobility. This greater mobility of the chain segments allowed more flexibility of the material and greater damping characteristics.

Moreover, it is well recognized that CNTs improve the dynamic response of carbon fiber reinforced composites according to the concept of the stick–slip mechanism as a consequence of the peculiar strong mechanical characteristics of CNTs [45,46,47]. For nanocomposites under applied stress, the load transfer from the polymer to nanotubes causes the deformation of both the matrix and the nanoparticles until a critical shear stress is reached. After this critical shear stress, the nanotube debonds from the polymeric matrix keeping its strain constant while the matrix continues to deform. In this “slipping” phase, there is no more load transfer and the structure dissipates energy because of the slippage between the polymeric matrix and the nanotubes, hence causing the damping [48].

#### Cone Calorimeter Test

**Figure 21:**
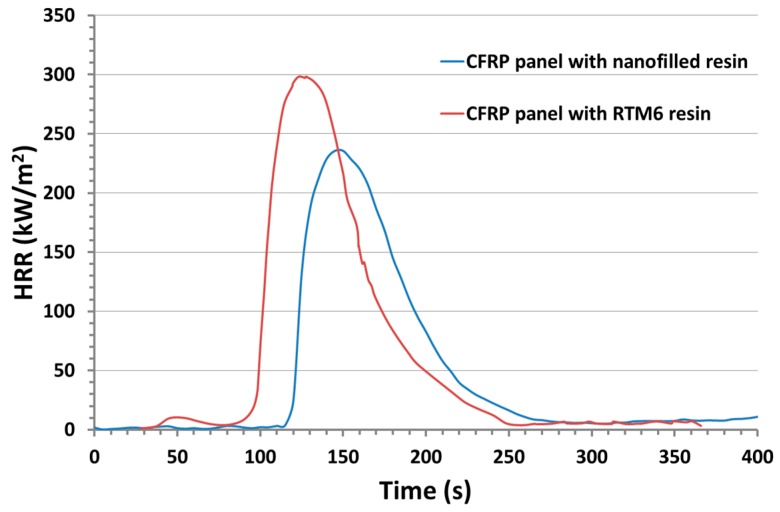
Heat release rate (HRR) of the composite obtained with the nanofilled resin to RTM6 panel.

The flammability of the manufactured panels was analyzed with the cone calorimeter and the results were compared to those obtained on panels made in the same conditions but using a commercially available epoxy resin. The heat release rate for both composites made with the RTM6 and nanofilled resins is shown in Figure 21. Compared to the traditional RTM6 resin, the panel with the epoxy nanofilled resin exhibited a significant improvement in fire resistance properties. In fact, for the panel impregnated with the nanofilled resin with respect to the panel manufactured using the RTM6 resin, a delay in ignition time in addition to an increase in the thermal resistance of the material, which was manifested as a significant delay and reduction of the heat release rate (HRR) peak and in Table 1, showed some of the results obtained with the cone calorimeter test performed at the irradiance of 35 KW/m^2^. Compared to the panel obtained with the commercial epoxy RTM6, the peak of heat release rate (PHRR), the average HRR, and total heat release (THR) of the panel impregnated with the nanocomposite formulation decreased from 301 kW/m^2^ to 236 kW/m^2^ with a reduction of 21.4%, from 118 kW/m^2^ to 61 kW/m^2^ with a reduction of 48.6% and from 20 MJ/m^2^ to 17 MJ/m^2^ with a reduction of 15%, respectively. These data highlight that the flammability of the panel with the nanocomposite formulation is strongly reduced.

**Figure 22:**
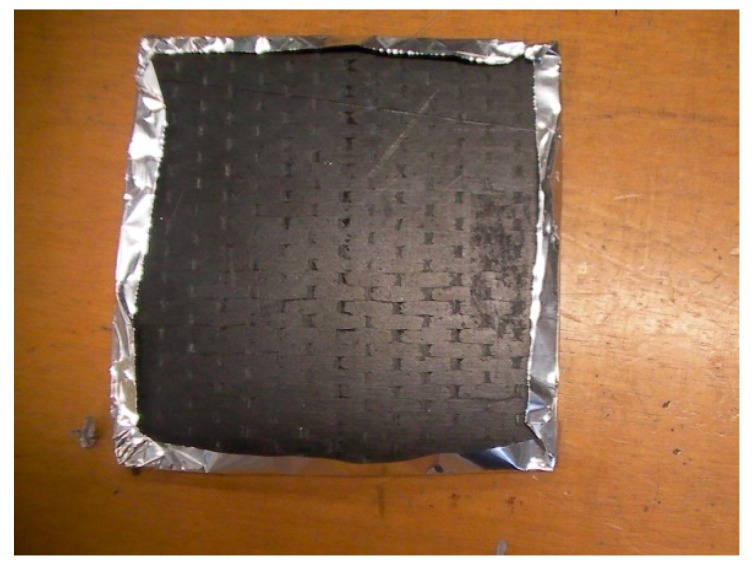
Photographs of the residue of the nanofilled panel after the cone calorimeter test performed at irradiance 35 kW/m^2^.

Figure 22 shows a picture of the sample after the cone calorimeter test. The non negligible presence of unburned resin is evident. The sample was almost self-extinguishing. This behavior may be due to the formation of non-intumescent char. The effect of the combined addition of GPOSS and MWCNTs in structural epoxy resin has already been studied by Raimondo et al. [32]. In their paper, the authors analyzed the results of the cone calorimeter test and made some consideration of the pictures of the residues after the test. An improvement of the fire performance was observed when any kind of POSS compound was included in the composite. Furthermore, the specific GPOSS was found to be able to promote the formation of intumescent char. The further inclusion of MWCNTs was causing, instead, a decreasing of the intumescence effect brought by GPOSS components, but the residue was found to be characterized by a more compact aspect highlighting the ability of MWCNTs to also promote char formation. 

The inhibition of the intumescent effect was attributed, in that paper, to the increase in thermal conductivity all over the sample but it may be also due to the increase of the viscosity due to the presence of CNT as highlighted in [33]. This increase in the viscosity could be also responsible for the increase in the time of ignition, as an increase of the viscosity inhibits the kinetics of the degradative phenomena and hence the velocity of the fuel formation in the gas phase. The char formation promoted by CNT is also well observable in Figure 22, for the carbon fiber panel manufactured in this work.

## 4. Conclusions

Different dispersion methods suitable for the incorporation of carbon nanotubes in an epoxy formulation containing solubilized GPOSS nanocages were investigated. The optimal method, consisting of a first step “Ultrasound radiation assisted by high stirring method” at cyclic pulse of 75%, the intensity of 100% for the ultrasound and of the use of the viscojet triple cone at 600 rpm for the mechanical high speed stirring, followed by a second step with the calendar for which the distance between roller was set at 2.5 mm, was used to manufacture a carbon fiber reinforced panel which was characterized both in terms of structural damping and of sound transmission loss properties. The new formulation demonstrated an improved efficiency with reference to a standard CFRP equivalent panel, showing modal damping over 3%, especially for the first normal modes and an STL value over 25 dB also in the low frequency range, with a very limited decrement in the normal modes area. Additionally, this circumstance gives evidence of the damping peculiarities. The obtained improvement of the modal damping and of the STL at low frequencies was explained both in terms of improved viscoelastic behavior of the polymeric nanofilled matrix, determining a greater mobility of the chain segments and consequently more flexibility of the material and using the concept of the stick–slip mechanism as a consequence of the peculiar strong mechanical characteristics of CNTs. 

The manufactured panel was also characterized in terms of fire performance using a cone calorimeter, and the results were compared to those obtained using a commercially available monocomponent RTM6 (Hexcel composites) epoxy aeronautic resin with the same process and the same fabric and lamination. Compared to the traditional RTM6 resin, the panel with the epoxy nanofilled resin exhibited a significant improvement in fire resistance properties both in terms of a delay in the ignition time and in terms of an increase in the thermal resistance of the material. The improvement of the fire performance was attributed to the formation of a non-intumescent char due to the simultaneous presence of GPOSS and carbon nanotubes.

It is well known that one of the weak points limiting the use of composites is their poor vibroacoustic performance. Due to their high stiffness to weight ratio, unlike metals, these materials vibrate in the same range of frequencies of the human auditory system. This affects the comfort of passengers, especially for large, long-distance cruise aircraft. The obtained improvement of the modal damping and of the STL at low frequencies together with the improved fire performance are encouraging, opening new perspectives in design and applicability of this material as structural materials also for the cabin of the aircraft.

## Figures and Tables

**Table 1:** Comparison of parameters by cone calorimeter test: time to ignition, tig, HRR results, and total heat release (THR) for measurements performed at irradiance 35 kW/m^2^.

Samples	t_ig_ (s)	tHRR_peak_ (s)	pHRR (kW/m^2^)	pHRR Delta %	HRR Average (kW/m^2^)	HRR Average Delta %	THR MJ/m^2^	THR Delta %
RTM6 panel	91	124	301		118		20	
Nanofilled panel	120	147.5	236.5	−21.43	60.7	−48.56	17	−15.00
